# Supplementary material for: Training Convolutional Neural Networks to Score Pneumonia in Slaughtered Pigs
Source: Animals (Basel). 2021 Nov 17;11(11):3290. doi: 10.3390/ani11113290 (PMC8614402; doi:10.3390/ani11113290)
Supplement: Supplementary file 1 [file animals-11-03290-s001.zip › File S1.pdf]

https://adal-demo.f4tlab.com/
